# Conservation, Duplication, and Divergence of Five Opsin Genes in Insect Evolution

**DOI:** 10.1093/gbe/evw015

**Published:** 2016-02-09

**Authors:** Roberto Feuda, Ferdinand Marlétaz, Michael A. Bentley, Peter W.H. Holland

**Affiliations:** Department of Zoology, University of Oxford, United Kingdom

**Keywords:** lepidoptera, arctiidae, butterfly, molecular evolution, adaptive evolution

## Abstract

Opsin proteins covalently bind to small molecular chromophores and each protein-chromophore complex is sensitive to particular wavelengths of light. Multiple opsins with different wavelength absorbance peaks are required for color vision. Comparing opsin responses is challenging at low light levels, explaining why color vision is often lost in nocturnal species. Here, we investigated opsin evolution in 27 phylogenetically diverse insect species including several transitions between photic niches (nocturnal, diurnal, and crepuscular). We find widespread conservation of five distinct opsin genes, more than commonly considered. These comprise one c-opsin plus four r-opsins (long wavelength sensitive or LWS, blue sensitive, ultra violet [UV] sensitive and the often overlooked Rh7 gene). Several recent opsin gene duplications are also detected. The diversity of opsin genes is consistent with color vision in diurnal, crepuscular, and nocturnal insects. Tests for positive selection in relation to photic niche reveal evidence for adaptive evolution in UV-sensitive opsins in day-flying insects in general, and in LWS opsins of day-flying Lepidoptera specifically.

## Introduction

The ability of animals to respond to visual stimuli is essential for many aspects of life. In insects, and more generally in Eumetazoa, this response is mediated primarily by opsins: a set of proteins belonging to the G-protein-coupled receptor (GPCR) superclass. Opsin proteins covalently bind a small molecule chromophore, typically derived from vitamin A, and together the complex is able to react to light. The chromophore undergoes photo-isomerization in response to a photon of light, inducing a conformational change in the opsin protein, and activation of a downstream signaling cascade. Most opsin genes are expressed in photoreceptors, but there are opsins expressed in other tissues suggesting some nonvisual functions (Terakita 2005; Shichida and Matsuyama 2009; Oakley and Speiser 2015).

Opsins originated early in metazoan evolution and duplicated to give three major gene families groups in bilaterians: c-opsins (ciliary opsins), r-opsins (rhabdomeric opsins), and RGR/Go opsins (including vertebrate peropsin) (Bellingham et al. 2003; Terakita 2005; Fain et al. 2010). The ciliary and rhabdomeric terminology reflects the structure of photoreceptor cells: in the former, the membrane of a cilium is folded to increase surface area for storage of opsin proteins, whereas in the latter the cell surface itself is deeply folded. Visual functions have rarely been found for RGR/Go opsins, although few have been studied (Gühmann et al. 2015). In general, c-opsins are probably the main visual pigments of vertebrates (including all opsins expressed in ciliary rods and cones of the eye), and r-opsins are used as the principal visual pigments in the light-sensitive membranes (rhabdomeres) of arthropod compound eyes (Porter et al. 2012). However, this long-assumed distinction between vertebrates and protostome invertebrates has been challenged in recent years and there is evidence for r-opsin expression in vertebrates and c-opsin use in protostomes (Arendt 2003; Arendt et al. 2004; Passamaneck et al. 2011).

Within the visual r-opsin family of insects, there are three widely accepted and well-studied paralogues: a long wavelength-sensitive opsin (LWS opsin, peak absorbance 500–600 nm), a blue sensitive opsin (Blue opsin, peak absorbance 400–500 nm), and an ultraviolet-sensitive opsin (ultra violet [UV] opsin, peak absorbance 300–400 nm) (Briscoe 2008; Yuan et al. 2010; Henze and Oakley 2015). In addition to this basic repertoire, an additional insect r-opsin paralogue has been identified in some species (Carulli et al. 1994; Attardo et al. 2014; Futahashi et al. 2015), named Rh7, for which the phylogenetic distribution is unresolved. These r-opsins are in addition to insect c-opsin, the evolutionary conservation of which is also unclear.

Opsin evolution cannot be understood without also considering the ecological interactions between animals and their environment. Color vision requires the expression of opsins with different spectral sensitivity, since an animal must compare the responses of two or more opsins tuned to different wavelengths of light (Osorio and Vorobyev 2008). In vertebrates, this is achieved through possession of multiple c-opsins, while insects use the multiple r-opsins described above; the fact that these expanded gene sets evolved by different gene duplications suggests that color vision evolved independently in vertebrates and insects. To some extent, the diversity of opsin genes present in a genome can give insight into the visual capability of the species. Possession of three visual opsins with different spectral sensitivity implies capacity for trichromatic vision, while a single opsin gene cannot provide color vision (Kelber and Roth 2006). It might be assumed that ability to see color would always be a trait favored over monochrome vision, but there are situations when the selection pressure to retain color vision might be relaxed. Nocturnal species or those inhabiting low-light environments such as caves tend to show reduced selective constraints on one or more opsin genes, or even gene loss (Tierney et al. 2015). Light availability seems to impose strong selective pressures on opsin sequence evolution (Kelber and Roth 2006; Osorio and Vorobyev 2008; Jacobs 2009; Zhao et al. 2009; Veilleux et al. 2013); for example, the LW and UV-sensitive opsins each show differences in the amino acids subject to strong selection between nocturnal and diurnal fireflies (order Coleoptera (Sander and Hall 2015). Similarly, a change in the spectral tuning of the LW opsin protein was also observed in adaptation to dim-light environment in augochlorine bees (Tierney et al. 2012).

In this study, we took a genome scale approach to examine the diversity of opsin genes in insects. We identified all opsin genes in 27 insect genomes (spanning seven orders); these included lepidopteran species (Ferguson et al. 2014) chosen to allow study of independent shifts from nocturnal to diurnal activity, such as the butterflies and the Arctiinae or “tiger moths.” We find a dynamic pattern of gene duplication and gene loss, but overall conservation of five opsin types across insects (LWS, Blue, UV, Rh7, c-opsin). The gene family distribution is consistent with color vision in diurnal, crepuscular, and nocturnal insects. Tests for positive selection in relation to photic niche revealed evidence for adaptive evolution in UV-sensitive opsins in day-flying insects in general, and in LWS opsins of day-flying Lepidoptera specifically.

## Materials and Methods

### Data Mining

Genome sequences for 20 insects covering seven orders were downloaded from Ensembl (Cunningham et al. 2015), plus the genomes of *Manduca sexta* (https://www.hgsc.bcm.edu/arthropods/tobacco-hornworm-genome-project, last accessed August 28, 2014) and *Plutella xylostella* (You et al. 2013). Each corresponding proteome was mined with BLASTp using the 449 opsin data set of Feuda et al. (2012). Consistent with previous work (Feuda et al. 2012) sequences with e-value <10^−^^10^ were retained, since this approach returns a diverse GPCR data set including all opsins plus other genes, which can then be further analyzed. To discriminate opsins from other GPCRs, we used a combination of a sequence similarity and motif analysis; to be retained as opsins, we required a top BLASTp hit with opsin in Uniprot and/or conservation of a recognizable retinal-biding domain. In addition, we used lower coverage genome sequences for *Polygonia c-album*, *Pararge aegeria*, *Callimorpha dominula, Cameraria ohridella*, and *Glyphotaelius pellucidus* (Ferguson et al. 2014) because these allow three independent shifts from nocturnal to diurnal lifestyle within a single order to be analyzed; since these genomes are low coverage and not annotated a different strategy was necessary. Reads were assembled using Velvet with k-mer sizes 31 and 41, and searched with tBLASTn (e-value <10^−^^10^) using opsins from *Apis mellifera*, *Drosophila melanogaster*, *M. sexta, Heliconius melpomene, Danaus plexippus*, and *Tribolium castaneum.* We assembled a final data set of 166 opsin sequences for analysis. Opsin intron/exon structure predictions were generated using a homology-based method with the program Geca (Fawal et al. 2012). Additionally, to gain a picture of the functional opsin number in each species, we predicted the number of transmembrane structure using Topcons (Tsirigos et al. 2015); opsin sequences predicted to have either six or seven transmembrane domains where retained as putative functional opsins .

Finally, photic niches (see below and supplementary table S1, Supplementary Material online) were inferred integrating personal knowledge of most species and information from the literature (Porter and Tschinkel 1987; Orr 1992; Zimmerman 1992; Kawada et al. 2005; Gentile et al. 2009; Rund et al. 2011; Marinotti et al. 2013). However, it should be noted that in some species such as *Aedes aegypti*, *Anopheles darling*, and *Atta cephalotes* there can be geographical or seasonal variation in behavior.

### Alignments and Phylogeny

Protein sequence alignment was performed in PRANK (Loytynoja and Goldman 2008), which has been shown to outperform other alignments methods with similar data (Loytynoja and Goldman 2008). The alignment was manually curated and indel-rich regions of uncertain alignment removed, and is available from Oxford University Research Data Archive (ORA-Data), under DOI 10.5287/bodleian:st74cq83q. Phylogenetic reconstruction was performed using PhyloBayes 3.3e (Lartillot et al. 2009) under the GTR-Γ (general time reversible-Γ) model (the best fitting model for large opsin data sets (Feuda et al. 2012, 2014). Trees were rooted using melatonin receptor (Fredriksson et al. 2003; Plachetzki et al. 2010; Feuda et al. 2012, 2014). Unrooted trees were also constructed to exclude potential error caused by using a distant outgroup. For all analyses, two independent runs were performed and convergence monitored using the maxdiff statistics calculated using the bpcomp program for PhyloBayes. Analyses were considered to have converged when maxdiff dropped below 0.3. Results of the analyses of the opsin + outgroup data sets were further tested by performing Maximum likelihood (ML) analyses under GTR-Γ (Stamatakis 2006).

### Positive Selection

To test for signatures of positive selection, the ratio of synonymous to nonsynonymous substitution rates (d_N_/d_S_ ratio or ω) was estimated in an ML framework using the CodeML program of PAML (Yang 2007). Codon alignments for each of the five opsin gene families were generated using PRANK with the codon option (Loytynoja and Goldman 2008; Markova-Raina and Petrov 2011). Ambiguously aligned residues and positions with greater than 60% gaps were removed. To test whether adaptation to diurnal or nocturnal lifestyle was accompanied by detectable adaptive sequence change, two data sets were analyzed. First, we included all insects; since inferring lifestyle of many ancestral nodes was not possible, we assigned ancestral states only when all descendants shared the character, with an undetermined state of character applied to the root. A branch-site model was applied allowing one d_N_/d_S_ ratio for diurnal species, one for nocturnal species, one for crepuscular species or species active in day and night, and one ratio for undetermined ancestral nodes (NSsites = 0 and mode = 2, gamma with four categories). Second, we focused specifically on Lepidoptera, with an ancestral nocturnal state assumed for the species under study, accompanied by three independent shifts to diurnal activity (butterflies, Tiger moth, *Cameraria*). Different d_N_/d_S_ ratios were allowed for nocturnal and diurnal. The Lepidoptera-only LWS opsin analysis was performed both including and excluding intron-less duplicated loci. We also undertook analysis to sites under selection associated with the d_N_/d_S_ increase in diurnal opsins by applying a branch-site model to the lepidopteran datasets (NSsites = 2 and model = 2, gamma with four categories). We considered the sites as candidates for positive selection according to the Bayes Empirical Bayes criterion. Sites deduced to be under selection were plotted onto 2D transmembrane topology inferred using Protter (Omasits et al. 2014).

## Results and Discussion

### Conservation of Five Opsins in Insect Evolution

**Fig. 1.—:**
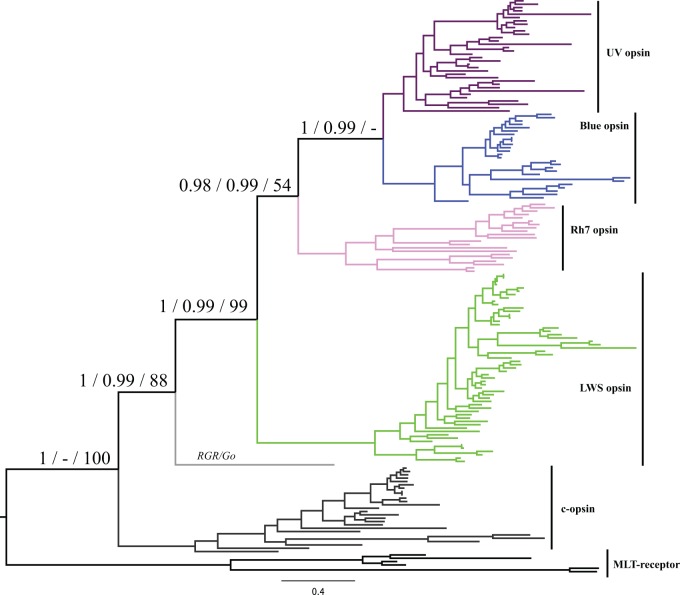
Phylogenetic tree of 166 opsins obtained using Phylobayes and a GTR-Γ model. On each node, the three support values shown are (left to right): PP of opsin + outgroup data set, PP of opsin unrooted data set, ML bootstrap of the opsin + outgroup data set. All phylogenetic trees were performed under GTR-Γ. The lack of support for the UV + Blue opsin clade in the ML tree is caused by phylogenetic instability of a single opsin from the human ectoparasitic louse (compare supplementary figs. S1–S3, Supplementary Material online). The orphan sequence not assigned to a named clade derives from pea aphid (*Acyrthosiphon pisum*). The same tree with species names is given in supplementary figure S1, Supplementary Material online.

**Fig. 2.—:**
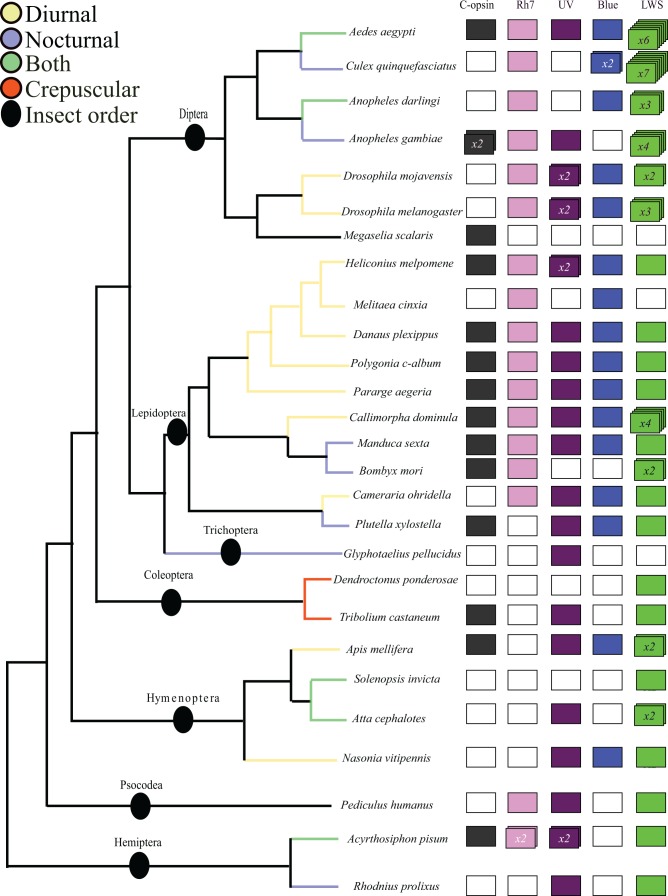
Opsin gene repertoire in genome sequences compared with a cladogram of insect evolution accordingly to Misof et al. (2014). When the number of genes identified is greater than one, this is indicated inside the rectangles. Additional genes are only accepted if the assembly predicts six or seven transmembrane domains; assignment deduced from phylogenetic analysis. White boxes indicate a gene is not found; this may reflect gene loss or genome incompleteness.

Phylogenetic analysis of all opsin sequences from 27 insect species confirms there are five major clades of insect opsin: one c-opsin and four r-opsins (LWS, UV, Blue and the enigmatic Rh7 opsin). All five opsins are very widespread across insect diversity (figs. 1 and 2; supplementary figs. S1–S3 and table S1, Supplementary Material online). Applying Bayesian phylogenetic analysis to an opsin plus outgroup data set, we find that UV and Blue opsins are sister clades (Posterior probability, PP, =1), which in turn form a monophyletic group with Rh7 opsins (PP = 0.98) and finally LWS opsins are the sister group to other r-opsins (PP = 1) (fig. 1, supplementary fig. S1, Supplementary Material online). An unrooted data set (supplementary fig. S2, Supplementary Material online) and an ML analysis (supplementary fig. S3, Supplementary Material online) recover essentially the same topology. Most studies of opsin diversity in specific insect orders have focused on just three opsins (LWS, UV, and Blue); our genome scale approach expands insect opsin diversity, as was also noted recently by Futahashi et al. (2015). The few studies undertaken suggest that the c-opsins in insects are not primarily visual, but are expressed in the brain and probably involved in circadian rhythms (Velarde et al. 2005). A dual role in vision and circadian entrainment has also been found for an insect r-opsin in an orthopteran insect (Komada et al. 2015). It is more difficult to assign a function to the Rh7 opsin, but we note that it is ancient in insects and very widespread; this conservation implies it certainly has functional relevance. In *Drosophila*, the gene is expressed in brain as well as retina (Papatsenko et al. 2001; Chintapalli et al. 2007; Graveley et al. 2011; Kistenpfennig (2012), while there is low level expression in adult dragonfly eyes (Futahashi et al. 2015). We suggest future studies of visual evolution in insects should not overlook this gene.

Combining the opsin phylogenetic analysis (fig. 1) with the species tree (fig. 2) implies that all four distinct r-opsin paralogues are ancient and date to early in insect evolution, or even prior to insect radiation (Misof et al. 2014). Together with recent data from dragonflies, order Odonata (Futahashi et al. 2015), this is consistent with the prediction of Briscoe and Chittka (2001) and Henze and Oakley (2015) that visual opsin diversity evolved very early in insect evolution and predates the origin of flowering plants approximately 150 Ma. Such a conclusion would imply that the complex color vision abilities of insects did not arise to adapt to the reflectance spectra of flowering plants, but rather the colors of fruit and flowers may have evolved to be more discernable to the preexisting color vision capabilities of insects (Briscoe and Chittka 2001; Osorio and Vorobyev 2008; Marshall and Arikawa 2014).

### Gene Duplication and Gene Loss in Insect Evolution

Recent work on dragonflies, order Odonata, a relatively basal clade within winged insects, identified two additional opsin genes that were either not found or not widespread in our study: RGR/Go and Arthropsin (Futahashi et al. 2015). We found one potential RGR/Go gene in the Hemiptera *Acyrthosiphon pisum*, although the sequence is divergent and identification is equivocal (supplementary table S1, Supplementary Material online). RGR/Go genes are found elsewhere in the animal kingdom and in a basal insect clade (Futahashi et al. 2015) so this clearly represents a gene loss in Holometabola. In most of the other cases where a particular opsin gene is missing from a species in our analysis, we cannot definitively conclude this is a gene loss, since few genome sequencing projects can claim completeness. The exception is absence of c-opsin in *Drosophila*; a particularly striking gene loss and one to which a high degree of confidence can be given.

We also found several intriguing cases of opsin gene duplication within the insects (fig. 2). Perhaps most interesting are the consistent duplications of LWS opsins in mosquitoes (with different numbers between species) and an extensive duplication of the LWS gene in a day-flying Scarlet Tiger moth *Cal. dominula* (Arctiidae; four genes). We find no cases as dramatic as the massive expansion of opsin gene numbers reported for dragonflies (Futahashi et al. 2015). The conclusion that the multiple LWS sequences identified in the Scarlet Tiger moth draft genome represent independent genes is further supported by analysis of intron positions (supplementary fig. S4, Supplementary Material online): three of the duplicate LWS genes of the *Cal. dominula* lack introns. It is not known if this reflects intron loss from tandemly duplicated loci or retroposition (integration of a DNA copy of an RNA transcript), although the latter mechanism would be intriguing since retroposed duplicates can only arise from germ-line expressed genes and would normally not copy regulatory sequences (Booth and Holland 2004). We note that intron-less opsin genes have evolved in other taxa, notably crustaceans, cnidarians, and cephalopods, with the current understanding that they function as visual photoproteins (Porter et al. 2007; Liegertová et al. 2015).

### Color Vision in Nocturnal Insects

The reduced amount of photons at night is thought to make the process of color vision difficult (Warrant and Dacke 2011). It had long been assumed that nocturnal insects use olfaction rather than vision to find and recognize flowers (Brantjes 1978). However, recent work on nocturnal Lepidoptera, notably Sphingidae or hawkmoths (Kelber et al. 2003), and on a species of nocturnal Hymenoptera (Warrant 2008), suggests that some night-flying insects are able to distinguish colors. Based on the distribution of opsin paralogues determined in the present study (fig. 2), we suggest that the majority of nocturnal insects are capable, at least potentially, of discriminating colors. It seems likely from these data that nocturnal Lepidoptera (e.g., *Pl. xylostella* and *M. sexta*) have trichromatic vision (fig. 2). Additionally, a crepuscular coleopteran (*T. castaneum*), a human ectoparasitic louse (*Pediculus humanus*), a nocturnal trichopteran (*G. pellucidus*), some nocturnal Diptera (e.g., *Anopheles gambiae*), and a nocturnal hemipteran (*Rhodnius prolixus*) have at least dichromatic color vision.

These findings imply that, unlike many nocturnal mammals, nocturnal and crepuscular insects have generally not lost the opsins that are maximally sensitive to high light levels. Although further species sampling would be beneficial, it seems nocturnal and diurnal insects have similar opsin repertoires.

### Adaptive Evolution of Opsins in Day-Flying Lepidoptera

**Fig. 3.—:**
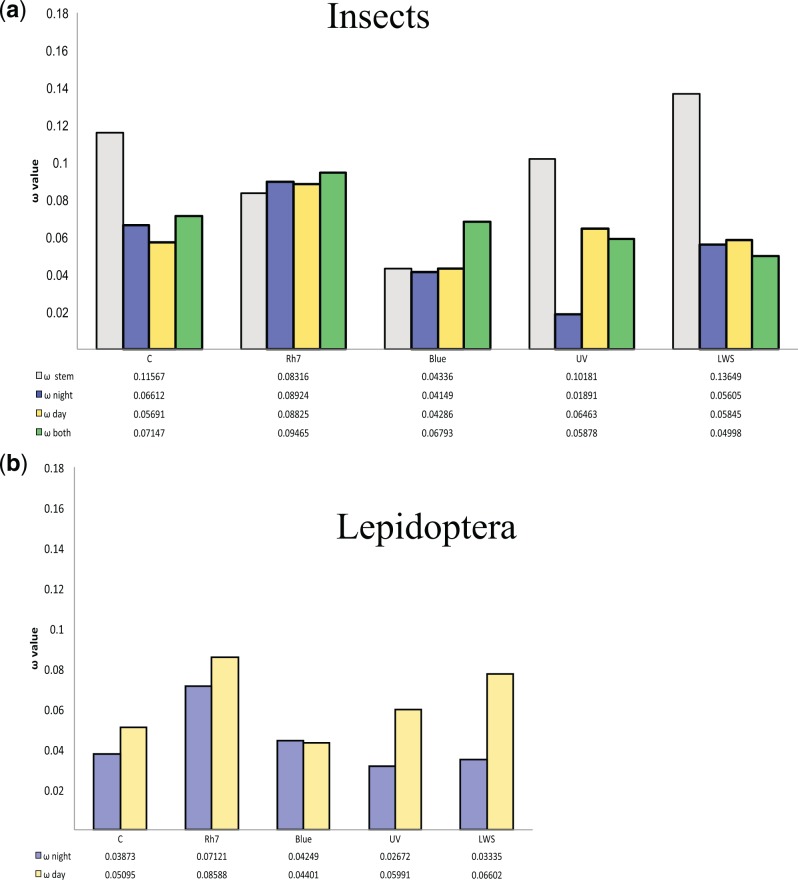
Pattern of positive selection inferred for the various opsin paralogues. (*a*) Comparison of ω for each paralogue across all insects, in relationship to lifestyle. (*b*) Comparison of ω for each paralogue across Lepidoptera, excluding intron-less *Cal. dominula* LWS sequences, assuming the common ancestor of the clade was nocturnal.

We asked whether nocturnal or diurnal lifestyles had driven adaptive sequence change in the five opsin genes. We first performed an analysis across all the insect species in the data set, making no assumptions about ancestral states where these are ambiguous. This revealed an increased global d_N_/d_S_ ratio (**ω** value), suggestive of adaptive protein sequence change, in the UV opsin gene of diurnal insects compared with nocturnal insects (0.065 vs. 0.019; fig. 3a and supplementary table S2, Supplementary Material online). Comparison to a single d_N_/d_S_ ratio model using a likelihood ratio test indicated that the multiple ratios have a better fit to the data (*P* < 0.0001). We repeated the same analysis focusing on Lepidoptera only, for which we defined the ancestral state as nocturnal. The majority of extant Lepidoptera are nocturnal, with shifts to diurnal lifestyle occurring in a few clades such as butterflies, tiger moths (Arctiinae), and some Leafminer moths (e.g., *Cam. ohridella*). This analysis mirrored the pattern detected for all insects, with the UV opsin having a d_N_/d_S_ ratio of 0.06 in diurnal lineages, compared with 0.03 in nocturnal lineages (fig. 3b, supplementary table S2, Supplementary Material online). Comparison to a single d_N_/d_S_ ratio model indicated that the two ratios have a better fit to the data (*P* < 0.0005). We speculate that the underlying adaptive reasons may be related to increased exploitation of UV-reflective patterns on flowers.

In addition, in Lepidoptera we detect a difference in d_N_/d_S_ ratio in the LWS opsins, with nocturnal moths having a ratio of 0.03 and this value increasing to 0.07 in diurnal lineages (fig. 3b and supplementary table S2, Supplementary Material online). This difference is detected whether we include or exclude the duplicated intron-less copies of LWS opsin from *Cal. dominula*. In each case, using different nocturnal and diurnal d_N_/d_S_ ratios fits the data better than a single ratio (*P* < 0.0001) (Tierney et al. 2012). Similar signatures of putative selection have been reported in LWS opsins of dim-light foraging sweat bees (Hymenoptera; Tierney et al. 2012) and in LWS and UV opsins of fireflies (Coleoptera; Sander 2015 #692}.

To examine if changes to d_N_/d_S_ ratio can be traced to positive selection in specific residues, we applied a branch-site model to the Lepidoptera UV and LWS opsin data sets, excluding the intronless *Cal. dominula* genes. We identified four and three amino acid sites, respectively, apparently under positive selection in the transitions from nocturnal and diurnal lifestyle (supplementary fig. S5, Supplementary Material online); several of these sites are located in the loops between transmembrane domains, suggesting they may be involved in opsin properties other than direct spectral tuning, unless the latter is mediated through long-range effects (Sander and Hall 2015). The adaptive reasons in Lepidoptera are unclear, but could conceivably be related to the extensive deployment of the color red in wing patterning of butterflies and diurnal tiger moths. In addition, if the absorption spectrum of LWS opsin extends into the very near-infrared, this may allow diurnal Lepidoptera to detect infrared scatter from healthy leaves (Gitelson et al. 1996) or the direction of incident sunlight for increasing body temperature prior to flight. Further comparative and experimental work will be needed to test these and other hypotheses. 

## Conclusions

We find that most insects possess five distinct opsin gene families, which have been subject to a variety of gene loss and duplication events. The phylogenetic distribution of the different opsin genes suggests that color vision may have evolved early in insects, before the origin of flowering plants. Furthermore, we suggest that the majority of nocturnal insects are able to discriminate colors. We identify a signature of adaptive sequence change in the UV opsin of diurnal insects, and the UV and LWS of Lepidoptera specifically. We speculate that the reasons for this positive selection may relate to wing color pattern recognition, host food-plant detection or body temperature control.

## Supplementary Material

Supplementary tables S1 and S2 and figures S1–S5 are available at *Genome Biology and Evolution* online (http://www.gbe.oxfordjournals.org/).

Supplementary Data
