# Supplementary material for: Conservation, Duplication, and Divergence of Five Opsin Genes in Insect Evolution
Source: Genome Biol Evol. 2016 Feb 9;8(3):579–87. doi: 10.1093/gbe/evw015 (PMC4824169; doi:10.1093/gbe/evw015)
Supplement: Supplementary Data [file supp_8_3_579__index.html]

Conservation, Duplication, and Divergence of Five Opsin Genes in Insect Evolution — Supplementary Data 

# Conservation, Duplication, and Divergence of Five Opsin Genes in Insect Evolution

## Supplementary Data

files

- Supplementary Data - zip file
