# Supplementary figures and images for: Conservation, Duplication, and Divergence of Five Opsin Genes in Insect Evolution
Source: Genome Biol Evol. 2016 Feb 9;8(3):579–87. doi: 10.1093/gbe/evw015 (PMC4824169; doi:10.1093/gbe/evw015)

Figure S1

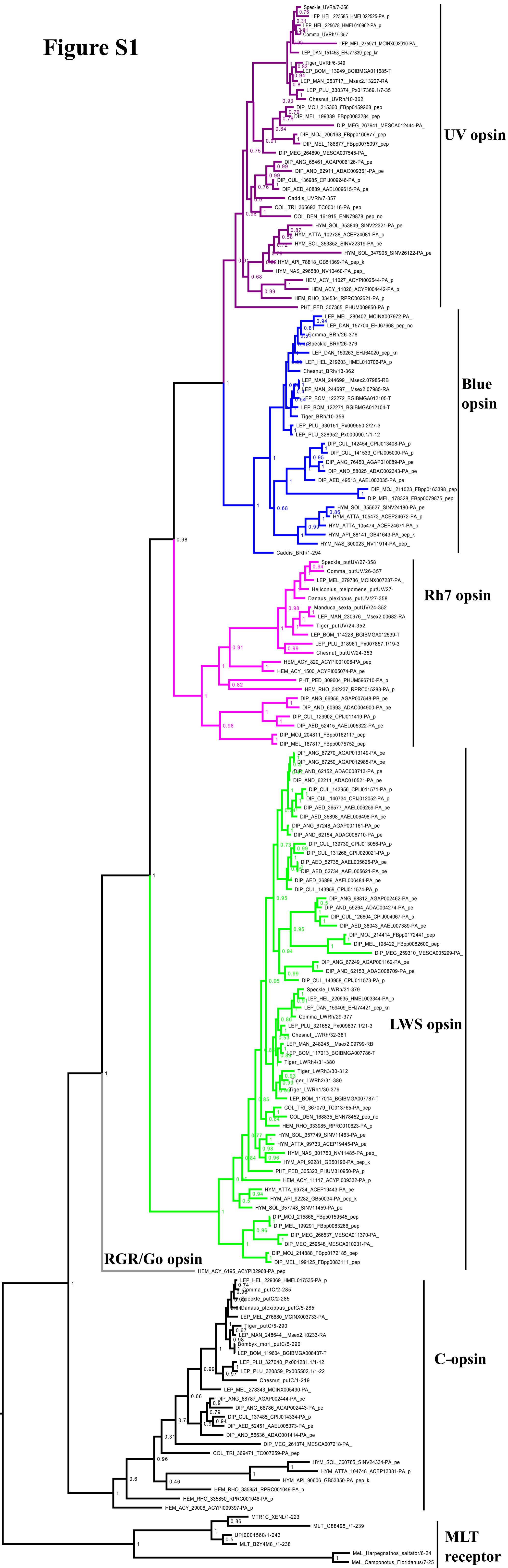

Supplement: Supplementary Data [file supp_evw015_suppl_data.zip › FigureS1.pdf]

Figure S2

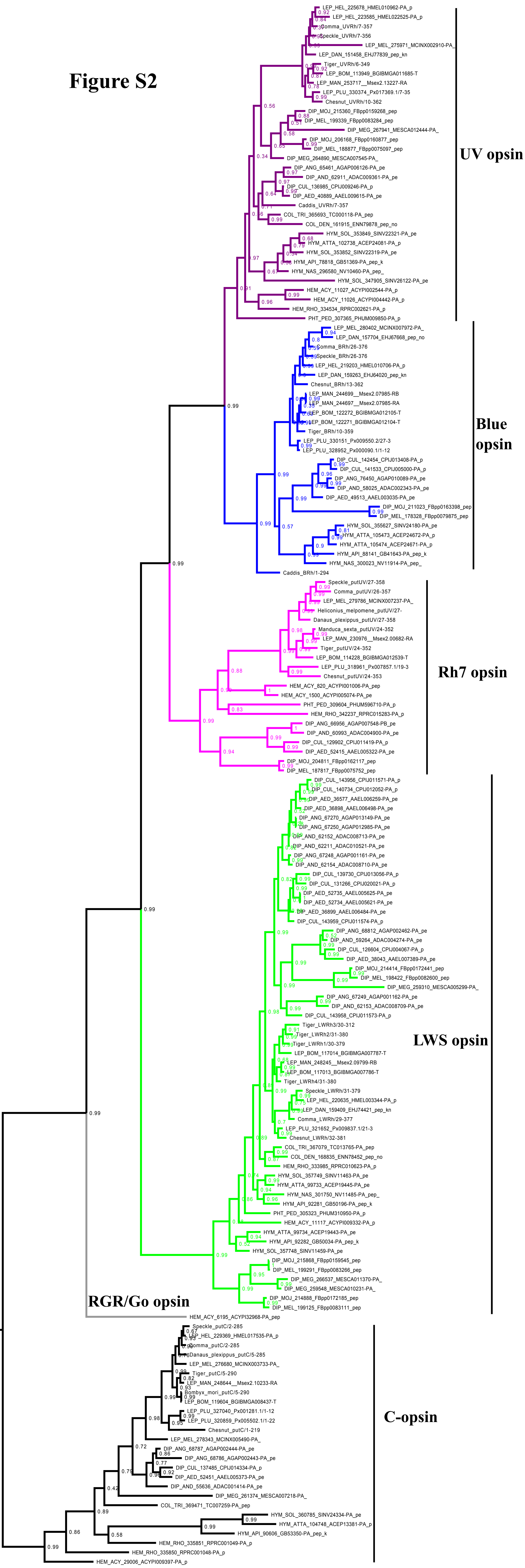

UV opsin

Blue opsin

Rh7 opsin

LWS opsin

C-opsin

Supplement: Supplementary Data [file supp_evw015_suppl_data.zip › FigureS2.pdf]

Figure S3

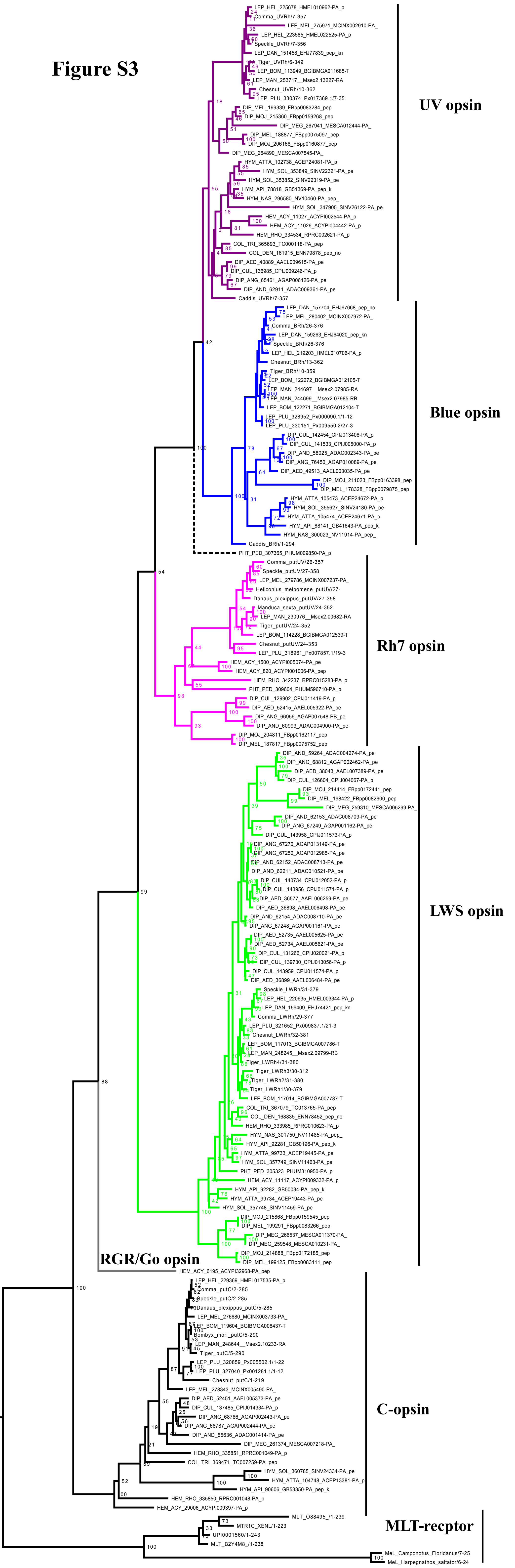

Supplement: Supplementary Data [file supp_evw015_suppl_data.zip › FigureS3.pdf]

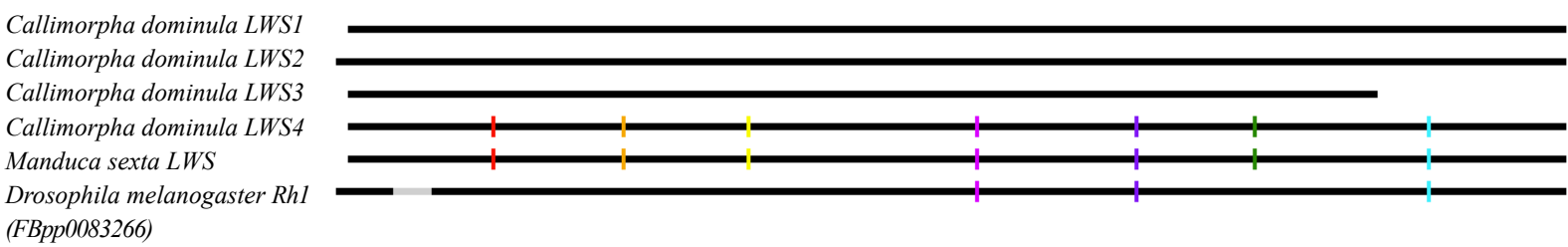

Supplement: Supplementary Data [file supp_evw015_suppl_data.zip › FigureS4_new.pdf]

# Figure S5

a

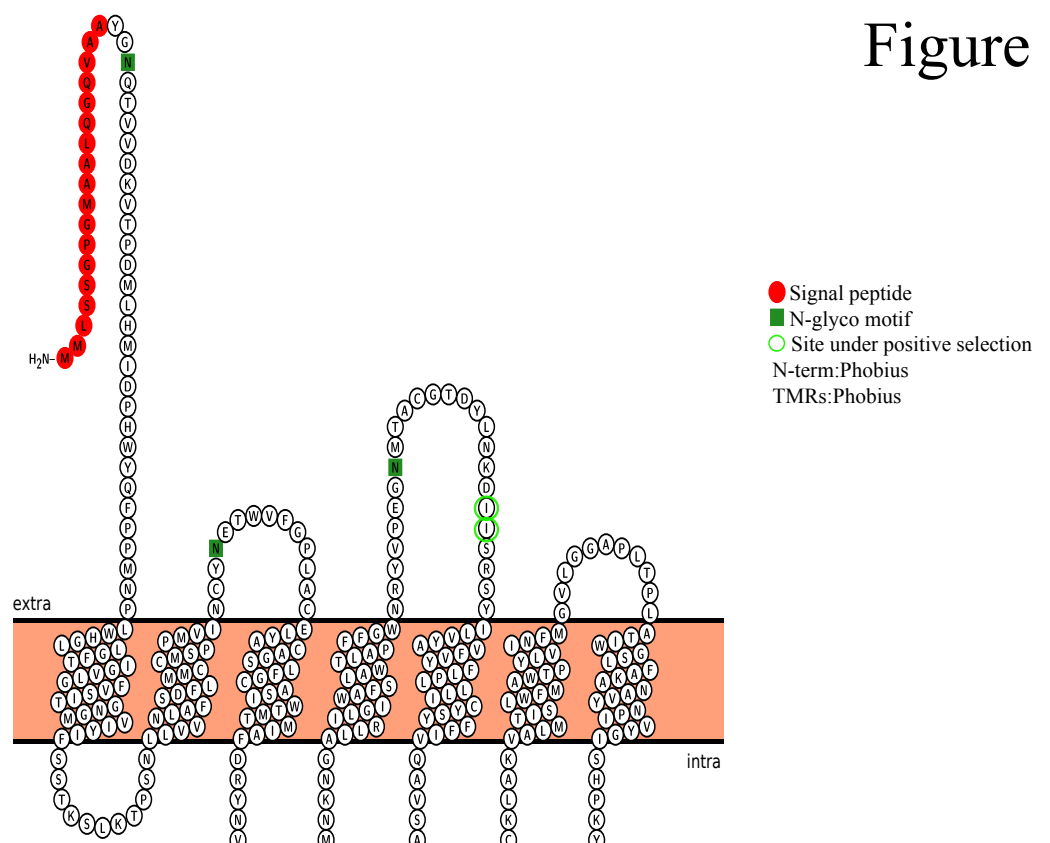

b

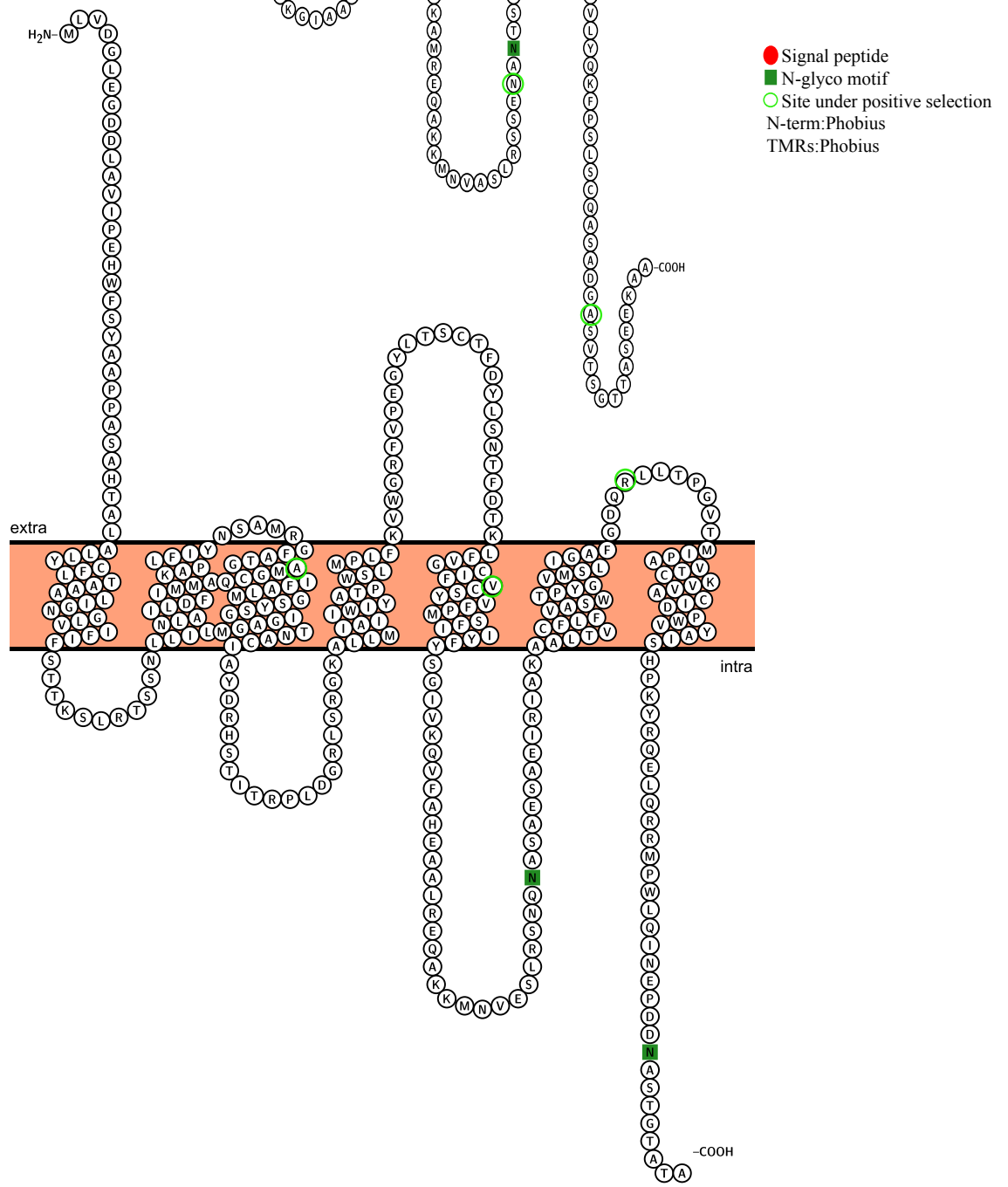

Supplement: Supplementary Data [file supp_evw015_suppl_data.zip › FigureS5_new.pdf]
